# The Science Behind Multidomain Interventions to Slow Cognitive Decline

**DOI:** 10.1093/geroni/igab046.258

**Published:** 2021-12-17

**Authors:** Laura Baker

**Affiliations:** Wake Forest School of Medicine, Winston-Salem, North Carolina, United States

## Abstract

The spotlight on interventions to protect brain health and prevent Alzheimer’s disease (AD) has recently widened to include risk modification. In the last 20 years, evidence continues to build to support cognition-enhancing effects of individual lifestyle components, which include, among others, physical exercise, diet, cognitive training, and cardiovascular risk management. A recent evolution of lifestyle trials is to combine these components as part of intervention delivery. The potential benefit of this approach on cognition in older adults, first showcased in the FINGER trial, is now under investigation by multiple groups across the nation and the globe. The multidomain approach offers important opportunities to boost lifestyle intervention ‘dose’, to examine inter-component synergistic effects, and for intervention tailoring to meet specific needs and limitations. Harmonization and data-sharing will be essential to meaningfully address the question of whether multidomain lifestyle modification can indeed be ‘medicine’ to protect brain health and reduce AD risk.

